# Combined activity-based therapy and transcutaneous spinal cord stimulation as an intensification and supplemental approach for upper limb sensorimotor recovery: a randomised controlled trial protocol for in-patients with subacute spinal cord injury

**DOI:** 10.1136/bmjopen-2026-117320

**Published:** 2026-08-14

**Authors:** Marika Demers, Nicolas Hoang Quang, Lucie Chesné, Sujata Sinha, Nancy Dubé, Héloïse Bourgeois, Diana Zidarov, El-Mehdi Meftah, Anne-Charlotte Desbarbieux, Julie Restout, Nok-Yeung Law, Amedeo Ceglia, Dorothy Barthélemy, CIME upper limb rehab team, Julie Comtois

**Affiliations:** 1 School of Rehabilitation, University of Montreal, Montreal, Quebec, Canada; 2 IURDPM, Centre for Interdisciplinary Research in Rehabilitation of Greater Montreal, Montreal, Quebec, Canada; 3 Neurosciences department, University of Montreal, Montreal, Quebec, Canada; 4 Biomedical Engineering, Polytechnique Montreal, Montreal, Quebec, Canada

**Keywords:** Spine, Neurological injury, REHABILITATION MEDICINE, Electric Stimulation Therapy

## Abstract

**Introduction:**

Activity-based therapy (ABT) delivers intensive, repetitive sensorimotor stimulation to enhance neuroplasticity after spinal cord injury. ABT has demonstrated synergistic benefits with cervical transcutaneous spinal cord stimulation (tSCS) for motor recovery particularly in individuals with chronic cervical spinal cord injury. However, its efficacy in subacute cases remains an emerging area of research. Accordingly, this study aims to determine the efficacy of an adjunctive ABT paired with cervical tSCS compared with ABT alone on upper limb function in people with cervical spinal cord injury receiving in-patient rehabilitation.

**Methods and analysis:**

This study is a single-centre, double-blind, two-arm randomised controlled trial. We will enrol 40 individuals with cervical spinal cord injury receiving in-patient rehabilitation (20/group). Participants will be randomly assigned to an intervention group (ABT+tSCS) or a control group (dose-matched ABT+sham stimulation). Both groups will receive 45 min of ABT+active or sham tSCS in addition to usual care for 20 sessions, 3 times per week. The primary outcome measures will be the Upper Extremity Motor Score of the International Standards for Neurological Classification of Spinal Cord Injury. Secondary measures will include clinical measures of upper limb impairments and activity limitations and neurophysiological measures (transspinal, motor and somatosensory-evoked potentials). All measures will be taken pre-intervention, post-intervention and at a 1-month follow-up.

**Ethics and dissemination:**

This study was approved by the Research Ethics Committee en réadaptation et en déficience physique of Santé Québec Centre-Sud-de-l’Île-de-Montréal - Universitaire (MP-2024-2080). All procedures will be conducted in accordance with the principles of the Declaration of Helsinki.

**Trial registration number:**

NCT07361627.

**STRENGTHS AND LIMITATIONS OF THIS STUDY**

- This study uses a rigorous control design with an active, dose-matched control group to isolate the specific effects of stimulation.
- Both participants and outcome assessors are blinded to group allocation to minimise bias.
- The study protocol was co-designed with individuals with lived experience of spinal cord injury, caregivers and clinicians to ensure clinical and personal relevance.
- Variability among participants with subacute spinal cord injury may introduce response variability and limit generalisability.

## Introduction

 More than 50% of injuries to the spinal cord occur at the cervical level (ie, tetraplegia), which consequently impacts arm and hand function.^1^ The arm and hand, collectively referred to as the upper limb, play a vital role in interacting with the environment. Following a spinal cord injury (SCI), the onset of spasticity, along with reduced strength, sensation and hand dexterity, limit participation and independence in daily activities and lead to a decreased quality of life.^2^ Regaining upper limb function is consistently identified as one of the most important priorities for people with tetraplegia.^2–4^ Unfortunately, there is a paucity of literature on the effect of training on upper limb function in people with cervical SCI.^5–8^

Activity-based therapy (ABT) is defined as any intervention that combines intense, repetitive and often rhythmical input to the central nervous system.^9^ It aims to stimulate neuromuscular changes through repeated activation of neuronal networks located above and below the injury and is considered an evidence-based intervention for people with SCI.^10 11^ ABT involves task-specific practice using external stimuli and cognitive functions, exercise progression and feedback provision, which can foster motor recovery.^12 13^ In a qualitative study, people with spinal cord injuries perceived that ABT can support neurological recovery, enhance physical and mental well-being, foster independence and instil hope.^14^

Although effective, ABT remains an indirect method of activating the nervous system. Recent advances in non-invasive electrical stimulation techniques enable the direct modulation of neuronal activity in the brain, spinal cord or peripheral nerves. This form of neuromodulation has demonstrated strong effects on neuroplasticity, leading to improvements in sensory, motor and autonomic functions, even in individuals with clinically complete SCI.^15–17^ Epidural stimulation can enhance voluntary movement amplitude in paralysed limbs and reduce spasticity in patients with incomplete SCI.^15 18–22^ However, its invasive nature and high cost have slowed down its translation into clinical practice.

In contrast, transcutaneous spinal cord stimulation (tSCS), a non-invasive approach, may target similar neural circuits and has demonstrated improvements in motor function.^21^ The mechanisms of action of tSCS are still not fully identified. One hypothesis is that spinal stimulation lowers the activation threshold of spinal motoneuronal pools, thereby enhancing the integration of residual descending supraspinal commands and segmental afferent inputs within spinal neuronal circuits to facilitate voluntary motor output.^21–23^ Recent evidence further shows that the effect of tSCS goes beyond the local spinal networks and extends into supraspinal neuronal networks.^24 25^ The posterior placement of tSCS electrodes relative to the spinal cord likely activates large-diameter sensory afferents near their dorsal root entry zones, providing robust afferent input to both spinal and supraspinal circuits. This stimulation may increase the excitability of sensorimotor networks, strengthen transmission through spared dorsal column pathways projecting to the primary somatosensory cortex (S1) and facilitate the integration of repeated sensory inputs arising from concurrent activity-based therapies.

Growing evidence supports the feasibility and potential efficacy of an approach combining ABT and cervical tSCS in individuals with long-standing SCI (chronic SCI).^26^ However, the integration of tSCS into clinical rehabilitation, particularly for individuals with subacute SCI, remains limited and understudied. To address this gap, the Neuromodulation and Intensification Research Clinic (CIME programme) was established at the Institut de réadaptation Gingras-Lindsay de Montréal (IRGLM)—one of only two specialised SCI rehabilitation centres in Quebec and the one serving the largest patient population. This research clinic was designed to implement early, intensive and personalised rehabilitation programmes that combine neuromodulation techniques, such as tSCS, with evidence-based ABT from admission (approximately 1-month post-injury) through the intensive rehabilitation phase. In that context, the upper limb project of the CIME programme is a randomised controlled trial (RCT) designed to compare the effect of cervical tSCS combined with ABT to an equivalent dose of ABT without stimulation in in-patients with subacute SCI. Individuals with SCI exhibit varying needs depending on the phase of recovery—acute, subacute or chronic—due to distinct pathophysiological processes and rehabilitation goals associated with each phase. The first few months after a SCI, when most intensive rehabilitation occurs, may capitalise on an enhanced window for experience-dependent plasticity^27^ to accelerate recovery and minimise maladaptive outcomes.

### Trial objectives

The overall objective of this RCT is to determine the effect of a supplemental intervention combining cervical tSCS+ABT on upper limb motor recovery, compared with supplemental ABT alone, in individuals with subacute cervical SCI undergoing standard in-patient rehabilitation. Participants will receive 20 intervention sessions, administered three times per week over 6–8 weeks, in addition to their usual care. Assessments will be conducted at baseline (pre-intervention), immediately post-intervention and at a 1-month follow-up. The primary outcome measure is the Upper Extremity Motor Score (UEMS) from the International Standards for Neurological Classification of Spinal Cord Injury (ISNCSCI), an established indicator of upper limb motor impairments.

### Secondary objectives

1. To determine the safety of the intervention (ie, minor and major adverse events (AEs)).
2. To determine changes in sensory and motor function measured by functional clinical assessments.
3. To explore the neuroplastic effects of the ABT+tSCS training programme on:
  - Spinal circuits, assessed via transpinal-evoked potentials (TEPs) using single-pulse tSCS;
  - Corticospinal pathways, assessed via motor-evoked potentials (MEPs) using transcranial magnetic stimulation (TMS);
  - Somatosensory pathways, assessed via somatosensory-evoked potentials (SSEPs) using electroencephalography (EEG).

We hypothesise that ABT+tSCS training will lead to greater improvements than ABT alone in the primary and secondary clinical and neurophysiological measures.

## Methods and analysis

### Design and setting

**Figure 1:**
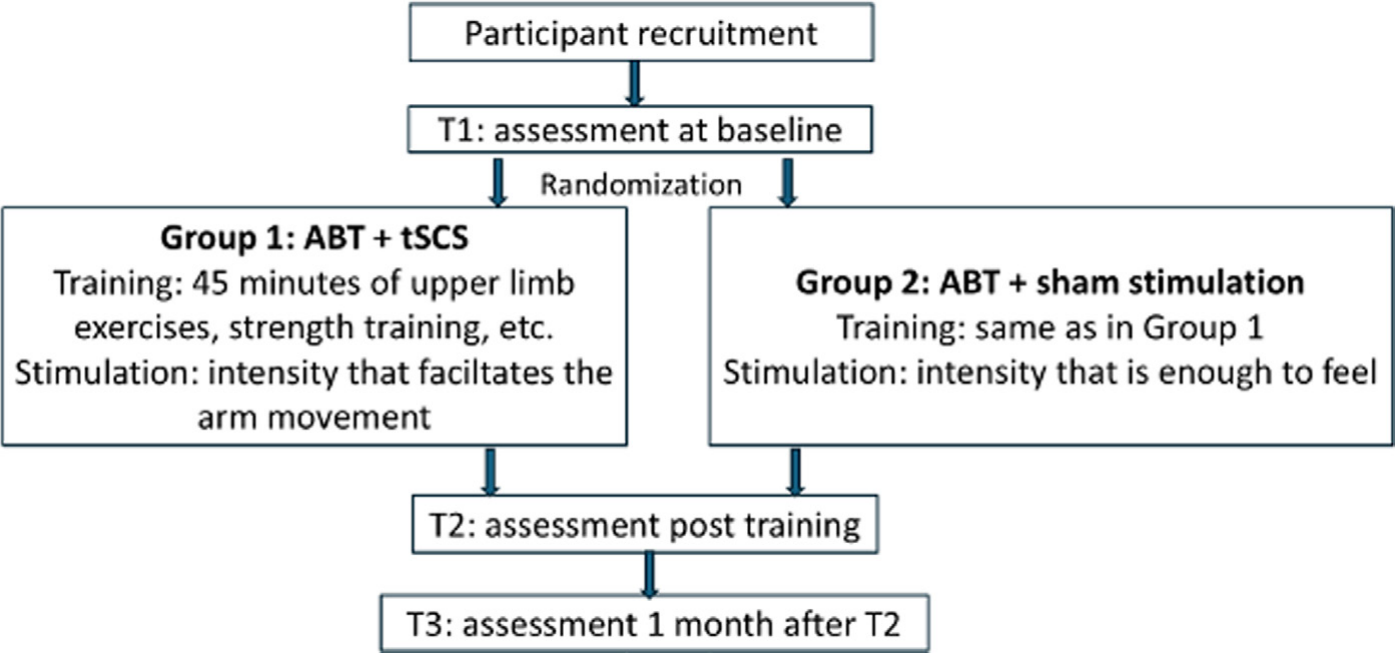
Flow diagram of Neuromodulation and Intensification Research Clinic (*CIME)* Upper Limb study with design and illustration of experimental and control groups’ allocation. ABT, Activity-based therapy; tSCS, transcutaneous spinal cord stimulation.

A single-centre, double-blind, two-arm RCT will evaluate the impact of the CIME programme (figure 1 displays the study flow). This study is part of a larger project aiming to co-develop and implement a Neuromodulation and Intensification Research Clinic for people receiving in-patient rehabilitation after a SCI.^28^ Participants will be randomly stratified and assigned to an intervention group (n=20) or control group (n=20). The intervention group will receive 20 sessions of tSCS combined with 45 min of active ABT over 6–8 weeks. The control group will follow the same treatment schedule but with sham (placebo) stimulation. Outcomes will be measured and analysed across these time points: before the training (T1), immediately after (T2) and 1 month following the end of the intervention (T3).

The data collection will take place at the IRGLM of Santé Québec Centre-Sud-de-l’Ile-de-Montréal - Universitaire, Québec, Canada. The IRGLM is a site of the Centre for Interdisciplinary Research in Rehabilitation of Greater Montreal and a 153-bed academic hospital specialising in rehabilitation for individuals with physical disabilities. As one of only two centres in Quebec specialising in SCI rehabilitation, IRGLM offers both in-patient and out-patient rehabilitation organised in two programmes (functional intensive rehabilitation and rehabilitation focused on social reintegration) and provides a strong foundation for feasible participant recruitment.

### Recruitment and consent

Participants will be recruited from the SCI units at the IRGLM from 1 September 2026 to 31 August 2028. Potential eligible participants will be identified by clinicians based on the inclusion and exclusion criteria (presented in table 1) and referred to the study team. Criteria will be reviewed by the research team. Given the haemodynamic instability frequently observed during the acute phase following SCI,^29^ some individuals may be ineligible for enrolment in the CIME trial, either temporarily or permanently. Clearance from the treating medical team will be obtained prior to enrolment.

**Table 1:** Inclusion and exclusion criteria

Inclusion criteria	Exclusion criteria
Traumatic and non-traumatic spinal cord injury	Cardiac pacemaker
Receiving in-patient rehabilitation	Active cancer at the stimulation site or metastatic cancer
Age≥16 years	Unstable lesion or clinical condition (eg, respiratory issues)
American Spinal Injury Association (ASIA) grade A–D	Electrode placement is impossible due to unhealed wound or significant local pain
Neurological level of injury between C5 and T2	Other neurological conditions (eg, stroke, Parkinson’s)
Residual upper limb motor impairments	Chronic liver or kidney disease
	Uncontrolled psychiatric condition
	Life expectancy<6 months
	Inability to provide informed consent (ie, severe cognitive impairment, dementia or Alzheimer’s disease)

Note: Participants with specific contraindications to transcranial magnetic stimulation, for example, epilepsy, unhealed skull fracture and increased intracranial pressure may participate in the study but will not take part in the transcranial magnetic stimulation assessment.

Once eligibility of the patient is confirmed, a clinician not involved in the research team will ask the patient whether they would be interested in hearing about a research project focusing on upper limb training. If the patient expresses an interest in hearing more about the study, a research coordinator will inform the patient of the nature of their participation and the risks and inconveniences and provide all information in oral and written form. Once the patient has given their written informed consent, they will be scheduled for their initial assessment session (see consent form in online supplemental material).

### Randomisation method and blinding

Forty participants who meet the inclusion criteria will be randomly assigned to either the intervention or control group. An independent statistician will generate the stratified randomisation sequence using Stata 16. Stratified randomisation with a 1:1 allocation ratio will be used to ensure balance across key characteristics, including age, sex and SCI severity as classified by the American Spinal Injury Association (ASIA). These variables were selected a priori because they are clinically important prognostic factors known to influence recovery and functional outcomes after SCI and imbalance across groups could bias treatment effect estimates. We acknowledge that the number of potential strata is relatively large compared with the target sample size (n=40), which may result in sparse strata. To address this, a restricted randomisation approach will be used (eg, permuted block randomisation). In addition, baseline characteristics will be carefully assessed, and any residual imbalance in key variables (particularly SCI severity) will be adjusted for in the primary analysis. This will be done in collaboration with the statistician on the team. This approach balances the need to control for important prognostic factors with the practical constraints of a small sample. Randomisation will occur after the initial assessment (T1). Neurological level and injury severity will be determined using the ISNCSCI,^30 31^ with impairment categorised according to the ASIA Impairment Scale, ranging from A (complete injury with no sensory or motor function preserved in sacral segments S4–S5) to E (normal function). Age will be categorised into three groups: 16–34 years, 35–49 years and 50+ years. Sex will be categorised into two groups: male and female. These variables were selected for stratification due to their potential influence on outcomes and to minimise confounding effects.^32–35^ To maintain balance in recruitment over time, permuted block randomisation will be employed with block sizes of 4 or 6. This approach will ensure equal distribution of participants between the intervention and control groups within each block, thereby reducing the risk of imbalance as the study progresses. The permutation of blocks will randomise the order of allocation, further enhancing the reliability and reproducibility of the randomisation process. A total of 40 participants will be included in the randomisation process to account for potential dropouts before the completion of the study (see section Power calculation). Consequently, 24 separate randomisation lists or *strata*, each containing 40 participants, will be generated based on the stratification variables. All study personnel involved in the clinical assessments will remain blinded to the group allocation. Participants will also remain blinded to their assigned group throughout the study. The interventionist will not be blind to the group allocation due to the immediate effect of tSCS.

### Assessments

#### Outcome measures

Participants will be assessed before the intervention (T1), post-intervention (T2) and at 1-month follow-up (T3) (see table 2). All clinical assessments will be administered by a trained occupational or physical therapist blinded to the group assignment. The same assessor will remain across all assessment time points.

**Table 2:** Primary and secondary outcome measures and assessment timepoints

Assessment	Timepoint			
	T1	Intervention	T2	T3
Primary outcome measures				
Upper-limb motor score of the ISNCSCI	x		x	x
Secondary clinical measures				
Prehension subtest of the GRASSP	x		x	x
Tetraplegic Upper Limb Activity Questionnaire	x		x	x
Grip and pinch strength	x		x	x
Upper-limb sensory score of the ISNCSCI	x		x	x
Monofilaments test	x		x	x
Proprioception subtest of the Fugl-Meyer Assessment	x		x	x
Finger-to-nose test	x		x	x
Neurophysiological measures (secondary)				
Transspinal-evoked potentials	x		x	x
Motor-evoked potentials	x		x	x
Somatosensory-evoked potentials	x		x	x

GRASSP, Graded Redefined Assessment of Strength, Sensibility and Prehension; ISNCSCI, International Standards for Neurological Classification of Spinal Cord Injury.

The clinical assessment battery will take approximately 2 hours to complete. All assessment results will be entered into the secure REDCap platform (Vanderbilt University, Nashville, TN), which complies with institutional and provincial data governance and confidentiality standards. Data entry will be performed by authorised personnel only, using coded identifiers to ensure participant confidentiality. All REDCap entries will be verified by another team member before locking the scores.

#### Primary measures

##### Primary outcome measure

*Upper limb strength:* The UEMS of the ISNCSCI aims to manually assess muscle strength at each metameric level using a scoring system from 0 to 5. Specific muscle actions are tested to represent each level: elbow flexion for C5, wrist extension for C6, elbow extension for C7, middle finger flexion for C8 and fifth finger abduction for T1. The scores range from 0 (no visible or palpable muscle contraction) to 5 (normal active movement, full range of motion against gravity and full resistance). The UEMS demonstrates excellent inter-rater reliability (intraclass correlation coefficient (ICC): 0.99) and a moderate to good validity (Spearman coefficient r=0.91).^36–38^

##### Secondary clinical assessments

*Upper limb activity limitation:* The Prehension subtest of the Graded Redefined Assessment of Strength, Sensibility and Prehension (GRASSP), V.2,^39^ is a standardised clinical outcome measure used to evaluate hand function through the assessment of grasp patterns and object manipulation abilities in individuals with cervical SCI. This subtest includes both qualitative and quantitative components. The qualitative component, called Prehension Ability, assesses the ability to perform three grasp patterns (eg, cylindrical, lateral pinch and tip pinch) based on the presence and quality of movement. Each hand is tested separately and scored from 0 to 4. A score of 4 indicates normal voluntary control of the wrist and digits when generating the respective grasp.

The quantitative component, Prehension Performance, involves functional tasks in which participants are asked to grasp, manipulate and release objects of varying shapes and sizes. Each hand is tested separately. The items are rated on a scale from 0 to 5. A maximum score of 5 indicates that the participant successfully solved the task using the expected grasp pattern. The GRASSP prehension has excellent test–retest reliability (ICC=0.93–0.98) and excellent inter-rater reliability (ICC=0.95–0.96).^39^

*Self-perceived upper limb function:* The Tetraplegic Upper Limb Activity Questionnaire (TUAQ) is a self-reported measure of upper limb function and satisfaction for individuals with tetraplegia. The TUAQ comprises 10 items, rated on a 5-point Likert scale ranging from 1 (not able to perform/not satisfied with the performance at all) to 5 (able to perform extremely well/extremely satisfied). Responses to each item are summed to obtain a raw score for each scale. This raw score is converted using a conversion table. The total score is between 0 and 100 for each scale, with a higher score indicating a higher performance or satisfaction. The TUAQ has good test-retest reliability for performance (ICC=0.89) and for satisfaction (ICC=0.88) and a high construct validity for TUAQ performance and satisfaction scales (rho: 0.19–0.52).^40^

*Grip and pinch strength:* The grip strength is evaluated with a JAMAR dynamometer, whereas the lateral, 2- and 3-point pinches are evaluated with a JAMAR pinch gauge. The participant sits with the elbow at 90 degrees, forearm neutral and wrist slightly extended and is asked to squeeze the device as hard as they can. The participant performs three trials for each pinch/grip type, and the average is recorded for each hand. There is an excellent inter-rater (ICC: 0.79–0.92) and excellent intra-rater reliability (ICC: 0.79–0.96).^41 42^

*Sensation:* Sensory function will be assessed using the ISNCSCI.^43^ Light touch and pinprick sensation will be examined bilaterally for dermatomes C4 to T1 at standardised sensory points on the upper limbs. Light touch will be tested using a cotton swab, and pinprick sensation will be assessed using a toothpick. Each dermatome and sensory modality is scored as 0 (absent), 1 (impaired or altered) or 2 (normal), based on comparison with sensation at an intact reference area. The sensory score of the ISNCSCI for the dermatomes from C4 to T1 detects the presence, alteration or loss of sensory function of spinal origin and assesses the participant’s ability to discriminate between light touch and sharp stimuli. The sensory function demonstrates excellent inter-rater reliability (ICC: 0.98–0.99) and moderate convergent validity (rho: 0.64–0.65).^37 38^

Tactile sensitivity will be assessed using the Semmes-Weinstein monofilament test, which measures cutaneous pressure detection thresholds through the application of calibrated nylon monofilaments applied perpendicularly to the skin surface. Testing will be conducted according to the Bell-Krokoski method,^44^ using a set of five monofilaments with diameters of 2.83, 3.61, 4.31, 4.56 and 6.65. Five anatomical sites will be tested on each upper limb: the pulp of the middle finger (D3; median nerve), the pulp of the little finger (D5; ulnar nerve), the first dorsal web space between the thumb and index finger (radial nerve), the lateral epicondyle over the extensor muscle compartment (lateral antebrachial cutaneous nerve) and the deltoid region (axillary nerve). Participants will be instructed to verbally indicate when the stimulus is perceived. The smallest monofilament detected at each site will be recorded, with lower scores indicating greater tactile sensitivity. The test has a moderate test–retest reliability (ICC: 0.46–0.61).^45^

*Proprioception:* The proprioception subtest of the Fugl-Meyer Assessment will assess the participant’s ability to locate their joint position in space at the shoulder, elbow, wrist and thumb. The evaluator performs four trials for each joint. The score ranges from 0 (absence) to 2 (correct 100%, little or no difference). For stroke survivors, there is an excellent inter-rater reliability (ICC: 0.93–1.0) and a good validity (r: 0.75),^46^ but this was not validated for SCI.

*Coordination:* The finger-to-nose test, performed based on the Comprehensive Coordination Scale,^47^ assesses smooth and coordinated upper limb movement. The participant is asked to alternately touch their nose and a target placed at arm’s length. The examiner scores four behavioural elements (trunk stability, arm movement, movement smoothness and endpoint accuracy) with a score ranging from 0 (impaired coordination) to 3 (normal) for each parameter. The time to complete five movement cycles is also recorded. The scores for each behavioural element are added for each side. The upper limb subscale of the Comprehensive Coordination Scale, developed for the stroke population, has excellent inter-rater reliability (ICC: 0.96) and intra-rater reliability (ICC: 0.96) and a moderate convergent validity (moderate–strong correlations with upper limb measures) in chronic stroke survivors.^47^

##### Neurophysiological assessment

Neurophysiological assessments will be conducted at T1, T2 and T3 by trained personnel with extensive experience in neurophysiological assessment to characterise neuroplastic changes in the central nervous system. TEPs induced by electrical stimulation will be used to assess changes in spinal neuronal networks. MEPs elicited by TMS will be used to assess corticospinal changes. For both methods, the responses to the stimulation will be recorded from the target muscles using electromyography (EMG). SEPs following median nerve stimulation will be recorded with EEG to assess sensory changes.

*Electromyography:* EMG activity will be recorded bilaterally from the following muscles, namely, biceps brachii, triceps brachii, extensor carpi radialis, flexor carpi radialis and first dorsal interosseous, using surface electrodes (Ag-AgCl, Blue sensor; interelectrode distance of 15–20 mm), secured to the skin over the belly of each muscle in accordance with SENIAM recommendations.^48^ EMG signals will be filtered (bandpass 10 Hz–1 kHz), amplified (500–1,000) and sampled at 2 kHz for online and offline analyses (CED Micro1401 with Signal software; Cambridge Electronic Design, UK).

###### Transspinal-evoked potentials

Single-pulse transcutaneous spinal stimulation is a non-invasive procedure used to assess the excitability of spinal pathways. Participants will remain comfortably seated in a chair or in their wheelchair. Self-adhesive surface electrodes will be placed over the cervical spine, with the cathode (Dura-Stick Plus double wire, Dura-Stick, US) positioned at the midline over C4-T1 and anodes over the external part of the clavicles (ROHVEMJ transcutaneous electrical nerve stimulation electrodes). Single-pulse tSCS will be delivered using a constant-current stimulator (DS8R, Digitimer, UK) with monophasic square-wave pulses of 1 ms at a frequency of 0.2 Hz to induce TEPs. Responses will be recorded from surface EMG. Stimulation intensity will be gradually increased to evoke a response in all, or the maximum possible number of, recorded upper limb muscles. The outcome measures will be: (i) motor threshold for each muscle, defined as the lowest intensity inducing 5 responses out of 10 stimulations; (ii) recruitment curve for each muscle and (iii) amplitude, latency and duration of the TEP at 120% of the motor threshold, determined for the myotome that showed the smallest response. Rest periods will be provided as needed to ensure participant comfort.

###### Transcranial magnetic stimulation

TMS will be used as a non-invasive approach to evaluate quantitatively the excitability of the corticospinal motor pathway. Single-pulse TMS (Magstim rTMS, Magstim Company Ltd, UK) will be applied using a figure-8 coil (90 mm outer diameter) over the arm area of the contralateral primary motor cortex while the subject will be comfortably seated in their wheelchair or the Magstim TMS chair. The optimal position (hotspot) for evoking MEPs preferentially in the bicep brachii muscle will be determined for both arms. The hotspot is defined as the scalp location that elicited MEPs of the largest amplitude in the bicep brachii muscle.^49–51^ This location is typically found lateral and anterior to the vertex, near the hand area of the primary motor cortex. The hotspot will be visualised on a computer through a neuronavigation system (Brainsight system; Rogue Research, Canada), which will ensure that the stimulation coil is maintained in the same position across all three assessment time points. The coil held by an experimenter will be positioned at a 45-degree angle relative to the midline.^52^ The active motor threshold (aMT) will be determined as the stimulus intensity at which 5 of 10 stimuli evoke a MEP larger than 100 μV of amplitude in the slightly contracted bicep brachii.^53^ Stimulus intensity will then be set at 120% of aMT, and 10 pulses will be delivered to characterise latency and amplitude of MEPs. A similar procedure will be performed to obtain the resting motor threshold (rMT) and characterise MEP during rest, where TMS will be applied without any contraction of the bicep brachii. The outcome measures will be the aMT and rMT for the targeted muscle. The latency, area and duration of the silent period (if present) will be also determined.

###### Somatosensory-evoked potentials

Participants will be comfortably seated in a chair or in their wheelchair. For each arm, the median nerve will be briefly stimulated using an electrode placed proximal to the wrist. This stimulation will be used to elicit SSEPs for each upper limb.^54–59^ A 32-channel EEG cap^60^ (Brain Vision Recorder 2.0, Brain Products GmbH, Gilching, Germany), with electrodes arranged according to the international 10-20 system,^61^ will be placed on the participant’s head to record cortical neuronal activity during the stimulation. During the 10-min EEG recording, median nerve stimulation will be delivered at a 3 Hz frequency for a total of 1800 stimuli. Each stimulus will last 0.001 s, with an inter-stimulus interval of 0.333 s. This repetitive stimulation will be delivered at an intensity corresponding to 5% of the maximal muscle response amplitude of the abductor pollicis brevis for each hand. The EEG will be recorded with the Fz electrode as the reference, as it is commonly used in the literature for upper limb stimulation.^57 58 62 63^ The sampling rate will be 5000 Hz.

In the post-recording phase, the raw EEG will be processed to generate the SSEPs using the BrainVision Analyzer 2.0 (Brain Products GmbH, Gilching, Germany), including filtering and averaging.^54 58 64^ Emphasis will be placed on the signals from the region of interest channels, C3/4 and their neighbouring electrodes (Cz, C1/2, Fcz, Cp3/4). The expected SSEPs are the N20 and the P25 that are consistently reported in SSEP studies done in SCI population.^65–68^ The N20 is a negative-going deflection of the baseline that peaks approximately 20 ms after the onset of the electrical stimulus at the median nerve.^67^ The P25, on the other hand, is a positive-going deflection that peaks around 25 ms from the onset of the stimulation, usually following the N20. In addition to the two primary early-latency SSEPs, the P50 and N70 components will also be explored.^69 70^ The P50 is a positive deflection peaking approximately around 50 ms following the median nerve stimulation onset, while the N70 is a subsequent negative deflection that peaks around 70 ms after stimulus onset. The outcome measures will be the absence or presence of SSEP. If present, the amplitude and the latency of the response will be measured to assess the excitability of the ascending somatosensory pathways.

### Intervention

#### Usual care

Usual rehabilitation care is delivered by a multidisciplinary team and focuses on maximising functional independence and reintegration into daily life. Typically, physical and occupational therapy sessions are delivered 4 times per week for 45 min per discipline, in addition to self-directed exercises. Occupational therapy sessions focus on developing physical capacity, self-care skills and mobility, whereas physical therapy sessions comprise strength-based exercises, balance and mobility exercises. The key principles of ABT are integrated in usual care. The possibility to participate in 1 hour of self-directed exercises is offered in a therapeutic gym equipped with a stationary bicycle and strengthening equipment. Individuals with SCI are also followed by a medical and psychosocial team comprised of a general practitioner, a physiatrist, a psychologist, a social worker and a nutritionist. Educational workshops are additionally offered to help participants better understand and manage their condition, promoting a holistic approach to rehabilitation. All participants will receive usual care in addition to the intervention, as per group allocation.

#### Training

ABT: Participants will receive 45 min sessions of active ABT+tSCS or sham, 3 times per week for 6–8 weeks, for a total of 20 sessions. The content of the ABT was developed using a participatory research approach with four occupational therapists and one physical therapist from the spinal cord programme at IRGLM, one patient-partner with C3-C4 tetraplegia and a caregiver.^71^ The ABT comprised five key sections: cardiovascular training, weight bearing and stretching and visually guided motor control training,^72 73^ strengthening and task-oriented training. The sequence of the ABT sections was designed to optimise neuromuscular activation, participant engagement and transfer of skills to real-life activities. Between sections, short breaks will be allowed to maximise engaged time-on-task. Time in active treatment will be recorded using a stopwatch to meet the 45 min target. Transitions between activities and rest periods during the task of more than 2 min will not be counted towards the total active time. Time targets are set for each ABT section, with a minimal duration for each ABT section (see table 3 for an overview of the time and content for each section). For participants with limited endurance, the minimal duration for each section will be offered in the first week and then will be progressively increased to meet the target duration by the end of week 3. The ABT protocol will be administered by a trained interventionist with a clinical background (physical or occupational therapy). Each participant will be paired with an interventionist to deliver the ABT protocol and a graduate student or research associate to apply the spinal cord stimulation (sham or active tSCS). The ABT protocol will be progressed to the needs of each participant. A collaborative approach will be used to select each activity. Participants will be encouraged to use their personal assistive devices (eg, universal cuff, adapted utensils) during task-oriented training.

**Table 3:** Section and time targets of the activity-based training session

Section	Target duration	Minimal duration	Key elements
Cardiovascular training	2 min	2 min	Modified jumping rope, Ergo-cycle (assisted or independent) or Vita-glide Moderate intensity (target of 4–5/10 on the modified Borg Perceived Exertion Scale)
Weight bearing and stretching	6 min	5 min	Weight-bearing on the forearm or the hand Stretches of the shoulder (flexion/extension, abduction, internal/external rotation), elbow (flexion/extension), wrist (flexion/extension) and fingers (flexion/extension, palm stretching, opposition)
Visually guided motor control training	6 min	3 min	Participants are instructed to make a cursor follow a series of target lines on a computer screen by performing voluntary flexion and extension movement within available range of motion guided by real-time joint angle feedback using an electrogoniometer (shoulder, elbow, wrist or hand)
Strengthening	8 min	6 min	Concentric, eccentric or isometric contractions of targeted group muscles (shoulder flexor or abductors, scapula, biceps, triceps, wrist extensors, pinches)
Task-oriented training	23 min	18 min	35 different tasks classified into seven categories: reaching, grasping, grasping with rotation, pinch, pinch with rotation, in-hand manipulation and finger isolation

#### Training: transcutaneous spinal cord stimulation

*Skin preparation and electrode configuration:* Prior to stimulation, the skin over the stimulated area will be cleaned with isopropyl alcohol. Once dried, the area will be prepared with Tape Skin Prep Electrode (Red Dot Trace Prep, 3M Canada) to reduce skin-electrode impedance. A single or two self-adhesive cathode electrodes (Dura-Stick plus double wire or ROHVEMJ transcutaneous electrical nerve stimulation electrodes) will be placed either along the midline or paravertebral region (still covering the midline), depending on potential significant asymmetries in the motor abilities of each participant. The electrodes will span the C4 to T1 vertebral levels at first to try and facilitate activity from any motoneuronal pool. In participants with better recovery, the electrode will only cover more caudal parts depending on what muscle needs to be facilitated. A pair of reference anodal self-adhesive electrodes will be placed bilaterally on the external part of the clavicular regions. All electrodes will be secured with elastic adhesive bandages (Tensoplast, BSN Medical-Essity, Sweden) to ensure optimal skin contact.

#### Experimental group

*Stimulation protocol:* Participants will receive tSCS throughout the ABT sessions. Stimulation will be delivered using a NeuroTrac Myoplus4 Pro (Verity Medical Ltd., UK). This stimulator provides the necessary range of intensity and frequency required, although the pulse duration is limited to 400 microseconds. Preliminary testing on able-bodied participants as well as SCI participants demonstrated that it could facilitate voluntary movement. Individualised tSCS parameters (30–80 Hz, 0–90 mA, biphasic 400μs pulse square) will be used to facilitate upper limb movement.

*Identification of stimulation parameters:* The effective stimulation parameters, frequency and intensity, will be determined prior to the first training session based on their ability to facilitate, if possible, a predefined voluntary movement. Facilitation is defined as an increased ease of movement reported by the patient accompanied by enhanced muscle activity, greater joint range of motion and/or improved motor control. This initial process will be guided by objective measurements using surface EMG, an electronic goniometer or visualisation of muscle twitch. First, electrode placement will be adjusted to ensure appropriate coverage of the upper limb myotomes targeted. Then, three frequencies will be tested based on what has been reported as efficient frequencies in the literature: 30 Hz, 50 Hz and 80 Hz.^21 74^ For each frequency, effective intensity will be defined as the lowest stimulation amplitude that reliably elicits facilitation without inducing pain or strong contractions that could hinder active training. Position and frequency will be kept constant throughout the training session but since physiological state and tolerance may vary from session to session, stimulation intensity will be reassessed at the beginning of each session. Reassessment will rely on visual observation to confirm the presence of facilitation. If the intensity required to elicit facilitation initially exceeds the participant’s tolerance, amplitude will gradually be increased during the session. If facilitation cannot be achieved at a tolerable intensity, it will be reported in the treatment notes and considered for interpretation of the effects. For each session, we will systematically document the muscles showing facilitation or visible twitches, the stimulation frequency and effective intensity used, the participant’s reported tolerance and any within-session parameter adjustments.

#### Control group

Participants assigned to the control group will follow the same procedure administered for participants in the experimental group for the ABT, except for the tSCS that will be delivered at sub-therapeutic intensity (2–3 mA). This level of stimulation will activate cutaneous afferents but remains below the threshold required to activate motor or sensory afferents.

### Data management and analysis

All study data will be collected, stored and managed in accordance with applicable ethical and regulatory standards to ensure participant confidentiality. Written informed consent forms will be kept in a secure, locked location within the clinical research facility. Data will be accessible only to authorised members of the research team. Questionnaire responses and results sheets will be stored as either paper or electronic records in REDCap. Hard-copy documents will be maintained in locked cabinets within the research facility, while electronic data will be anonymised and stored on password-protected computers in a secure environment. In compliance with the research centre’s data retention policy, all study data will be securely archived 7 years following study completion.

### Statistical analysis

The Shapiro-Wilk test will be used to assess the distribution of continuous variables for both demographic and outcome measures. Normally distributed continuous data will be summarised as mean with SD, while non-normally distributed data will be presented as median with IQR. Categorical variables will be reported as frequencies and percentages. Specific injury-related characteristics (eg, level and severity of injury) will be considered for exploratory subgroup analyses. To evaluate changes over time and differences between groups, a linear mixed-effects model will be fitted with fixed effects for group (ABT+tSCS and ABT+sham), time (pre, post and 1-month follow-up) and the group×time interaction and a random effect for participant to account for within-subject correlations across repeated measurements. The primary effect of interest will be the group×time interaction, which will be used to determine whether changes over time differ between groups. Where a significant group×time interaction is observed, estimated marginal means will be compared using post hoc pairwise contrasts with appropriate adjustment for multiple comparisons. Missing data will be handled using an intention-to-treat approach, with all available observations included in the mixed-effects model. If warranted, sensitivity analyses using multiple imputation may also be conducted to assess the robustness of the findings. The threshold for statistical significance will be set at p<0.05 for all analyses.

#### Power calculation

An a priori power analysis (G*Power 3.1) for a two-group, three-time-point repeated-measures ANOVA on the group×time interaction (α=0.05, two-tailed) indicates the following: detecting a moderate interaction effect (Cohen’s f=0.25; ≈ηp²≈0.06) requires N≈34 total (≈17/group) for 80% power. Allowing for~15% attrition over follow-up, we will recruit n=40 (20 per arm) to ensure ≥80% power to detect a moderate effect. The target effect size is justified by prior SCI neuromodulation studies showing moderate-to-large gains in hand/arm outcomes.^75^

##### Project management

*Daily Management:* Project oversight will occur at multiple levels. A weekly 15-min scrum meeting between the clinical and research leads will allow close monitoring of project progress and identification of barriers or facilitators to its smooth implementation. The leads will report to the CIME Programme research coordinators, who meet weekly with the principal investigators to discuss updates, issues or notable changes. The principal investigators will also meet semi-annually (or more frequently if required) with the managers of the SCI units to review project activity, resource use and any emerging concerns. In parallel, the project team will provide weekly updates to the SCI physiatrists to ensure they are informed about participant involvement and can interact rapidly with the research team if needed—particularly important since the intervention occurs early after injury. The research team will also meet weekly with the infection-control officer to confirm adherence to all safety protocols. In the event of a nosocomial or other infection, appropriate personal protective equipment (eg, gloves, masks and gowns) will be used to ensure the safety of both participants and staff.

*Training of research personnel:* To ensure the consistency of training with several therapists and clinicians involved, a structured training process, including multiple training sessions, mentoring and assessment of the fidelity of the intervention, will be implemented to maintain data quality and ensure uniform intervention delivery among all CIME interventionists. The training will include theoretical instructions, a detailed review of each section of the ABT protocol for clinicians and proper placement, parameter setting and assessment of facilitation effects for tSCS technicians. A detailed manual of procedures will be written and used. Regular meetings with all therapists will be held quarterly to discuss any outstanding issues and ensure adherence to intervention protocol. Ongoing supervision and periodic assessment will ensure consistency and fidelity throughout the trial.

##### Safety and adverse events

###### Data Safety and Monitoring Board

A Data Safety and Monitoring Board (DSMB) will be established to oversee participant safety and ensure the scientific and ethical integrity of the study. The DSMB will comprise the head physiatrist of the SCI unit at the research centre, the research coordinators and an independent researcher with expertise in clinical trials. The DSMB will convene every year or on an ad hoc basis if safety concerns arise. Its responsibilities include monitoring participant safety through annual reviews of AEs and unanticipated problems, ensuring the study is conducted ethically and with scientific rigour, and—if necessary—recommending modifications to address any raised issues. Recommendations may be based on evidence of significant adverse effects in the experimental group or on ethical considerations.

###### Adverse events

AEs will be closely monitored at each assessment time point and during all intervention sessions using a standardised AE monitoring form. The skin at the stimulation site will be routinely checked for irritation or redness. At the start of each session, participants will be asked about any changes in their medical status since the previous visit. An AE is defined as any undesirable or unfavourable medical occurrence in a participant—such as abnormal signs, symptoms or diseases—that arises during the study, regardless of whether it is considered related to the research. AEs may include new conditions emerging after enrolment or the worsening of pre-existing conditions. Each AE will be classified according to three dimensions: (1) seriousness: serious (life-threatening, requiring or prolonging hospitalisation or causing significant disability) or non-serious (transient and mild, with no lasting consequences); (2) causality: related or unrelated to study participation and (3) expectedness: expected or unexpected.

It is possible that if skin redness occurs, a hypoallergenic moisturising cream will be applied, with an in-person follow-up at 24 hours. If redness persists, electrodes will not be placed on the same location during the next session. If an AE occurs during the intervention sessions, the interventionist and stimulation operator will jointly assess any AE to determine its severity and appropriate response. For minor events, the session will be stopped and the participant returned to their room. The SCI unit nurse will be informed to document the incident in the patient’s chart, followed by next-day follow-up before resuming training. For more severe events, an emergency code will be activated according to institutional procedures. This standardised protocol mobilises a rapid-response team of clinicians within approximately 2 min to provide immediate medical assessment and care. Subsequent discussion with the treating physician will determine when/if training can safely resume. In all cases, the principal investigator will be notified, and the event will be documented in accordance with ethics and institutional requirements. All AEs will also be recorded in the REDCap database, ensuring compliance with ethics committee reporting procedures.

## Ethics and dissemination

This study was approved by the Institution Review Board (IRB) Committee en réadaptation et en déficience physique of Santé Québec Centre-Sud-de-l’Île-de-Montréal - Universitaire (MP-2024-2080) and is registered on clinicaltrial.gov. All procedures will be conducted in accordance with the principles of the Declaration of Helsinki. Any modifications to the protocol will be submitted to the IRB for approval.

Findings from this trial will be submitted for publication in peer-reviewed journals and presented at national and international conferences, such as the International Spinal Cord Society or the ASIA. A summary of the research results will be produced and distributed to all participants and clinical partners at the end of the study.

## Supplementary material

10.1136/bmjopen-2026-117320 online supplemental file 1
