# Supplementary material for: Combined activity-based therapy and transcutaneous spinal cord stimulation as an intensification and supplemental approach for upper limb sensorimotor recovery: a randomised controlled trial protocol for in-patients with subacute spinal cord injury
Source: BMJ Open. 2026 Aug 14;16(8):e117320. doi: 10.1136/bmjopen-2026-117320 (PMC13479523; doi:10.1136/bmjopen-2026-117320)
Supplement: Supplementary file 1 [file bmjopen-2026-117320.reviewer_comments.pdf]

PEER REVIEW HISTORY

BMJ Open publishes all reviews undertaken for accepted manuscripts. Reviewers are asked to complete a checklist review form and are provided with free text boxes to elaborate on their assessment. These free text comments are reproduced below.

ARTICLE DETAILS

Title (Provisional)

Combined activity-based therapy and transcutaneous spinal cord stimulation as an intensification and supplemental approach for upper limb sensorimotor recovery: a randomized control trial protocol for in-patients with subacute spinal cord injury

Authors

Demers, Marika; Hoang Quang, Nicolas; Chesné, Lucie; Sinha, Sujata; Dubé, Nancy; Bourgeois, Héloïse; Zidarov, Diana; Meftah, El-Mehdi; Desbarbieux, Anne-Charlotte; Restout, Julie; Law, Nok-Yeung; Ceglia, Amedeo; Barthélemy, Dorothy; CIME , upper limb rehab team

---

VERSION 1 - REVIEW

**Reviewer**	**1**
**Name**	**Ganau , Mario**
**Affiliation**	**University of Toronto**
**Date**	**05-May-2026**
**COI**	**None**

This is an excellent trial on rehab strategies for SCI patients, I have noticed that the reference 8 on the guidelines for timing of decompression is outdated and i would recommend to change with the 2024 guidelines:

- Fehlings MG, Tetreault LA, Hachem L, et al. An Update of a Clinical Practice Guideline for the Management of Patients With Acute Spinal Cord Injury: Recommendations on the Role and Timing of Decompressive Surgery. Global Spine J. 2024;14(3_suppl):174S-186S. doi: 10.1177/21925682231181883.

Also given the issues related to haemodynamic management after SCI which may well affect the start of early physiotherapy and rehabilitation process I would suggest including a sentence about this barrier and potential cause of ineligibility for neuromodulation. A good reference from the same body of recommendations is:

- Evaniew N, Davies B, Farahbakhsh F, et al. Interventions to Optimize Spinal Cord Perfusion in Patients With Acute Traumatic Spinal Cord Injury: An Updated Systematic Review. Global Spine J. 2024;14(3_suppl):58S-79S. doi: 10.1177/21925682231218737.

**Reviewer**	**2**
**Name**	**Minetama, Masakazu**
**Affiliation**	**Wakayama Medical University Kihoku Hospital, Spine Care Center**
**Date**	**05-Jun-2026**
**COI**	**None**

Thank you for the opportunity to review this interesting protocol manuscript investigating the efficacy of combining activity-based therapy (ABT) with transcutaneous spinal cord stimulation (tSCS) for upper limb recovery in individuals with subacute cervical spinal cord injury. The study addresses an important clinical question because most previous evidence has focused on chronic SCI populations, whereas the potential benefits of neuromodulation during the subacute rehabilitation phase remain unclear. The protocol is generally well written, clinically relevant, and includes rigorous elements such as assessor blinding, sham stimulation, and comprehensive neurophysiological assessments. The integration of patient partners in protocol development is also a notable strength. This reviewer has several questions that should be addressed prior to publication.

The abstract identifies both the UEMS and Grasp and Release Test as primary outcomes, whereas the methods section later refers to UEMS as the primary outcome measure. Please clarify whether there is one primary outcome or two co-primary outcomes.

The planned stratification by age (three categories), sex, and AIS grade may generate a large number of strata relative to the proposed sample size (n=40). This could result in sparse strata and reduce the effectiveness of the randomization procedure. The authors should provide a rationale for this approach or consider reducing the number of stratification variables.

The proposed statistical analysis requires further clarification. Although repeated-measures ANOVA is specified as the primary analysis, the protocol also states that missing data will be handled using multiple imputation or mixed-model estimation. Given the likelihood of missing data in a longitudinal trial, a mixed-effects model may be more appropriate than repeated-measures ANOVA. The authors should clarify the primary analytical approach, the strategy for handling missing data, and whether both intention-to-treat and per-protocol analyses will be conducted.

VERSION 1 - AUTHOR RESPONSE

Reviewer: 1

R1.1. This is an excellent trial on rehab strategies for SCI patients, I have noticed that the reference 8 on the guidelines for timing of decompression is outdated and i would recommend to change with the 2024 guidelines:
- Fehlings MG, Tetreault LA, Hachem L, et al. An Update of a Clinical Practice Guideline for the Management of Patients With Acute Spinal Cord Injury: Recommendations on the Role and Timing of Decompressive Surgery. Global Spine J. 2024;14(3_suppl):174S-186S. doi: 10.1177/21925682231181883.
Response R1.1: Thank you for pointing out that a more recent version of the clinical practice guidelines for management of SCI was available. We modified the reference #8.

R1.2. Also given the issues related to haemodynamic management after SCI which may well affect the start of early physiotherapy and rehabilitation process I would suggest including a sentence about this barrier and potential cause of ineligibility for neuromodulation. A good reference from the same body of recommendations is:
- Evaniew N, Davies B, Farahbakhsh F, et al. Interventions to Optimize Spinal Cord Perfusion in Patients With Acute Traumatic Spinal Cord Injury: An Updated Systematic Review. Global Spine J. 2024;14(3_suppl):58S-79S. doi: 10.1177/21925682231218737.
Response R1.2: This is a valid concern. Thank you for the reference. In the recruitment and consent section, we added two sentences on the haemodynamic instability: 'Given the hemodynamic instability frequently observed during the acute phase following SCI (Evaniew et al. 2024), some individuals may be ineligible for enrollment in the CIME trial, either temporarily or permanently. Clearance from the treating medical team will be obtained prior to enrolment.'

Reviewer: 2

R2.1. Thank you for the opportunity to review this interesting protocol manuscript investigating the efficacy of combining activity-based therapy (ABT) with transcutaneous spinal cord stimulation (tSCS) for upper limb recovery in individuals with subacute cervical spinal cord injury. The study addresses an important clinical question because most previous evidence has focused on chronic SCI populations, whereas the potential benefits of neuromodulation during the subacute rehabilitation phase remain unclear. The protocol is generally well written, clinically relevant, and includes rigorous elements such as assessor blinding, sham stimulation, and comprehensive neurophysiological assessments. The integration of patient partners in protocol development is also a notable strength. This reviewer has several questions that should be addressed prior to publication.

The abstract identifies both the UEMS and Grasp and Release Test as primary outcomes, whereas the methods section later refers to UEMS as the primary outcome measure. Please clarify whether there is one primary outcome or two co-primary outcomes.
Response R2.1: This is an oversight from our part. The UEMS is the primary outcome measure. We have revised one of the secondary clinical measures to the Prehension test of the Graded Redefined Assessment of Strength, Sensibility and Prehension (GRASSP) instead

of Grasp and Release Test due to the floor effect observed in our feasibility study. We modified the abstract accordingly and removed the mention of the Grasp and release test as a primary measure in the abstract. We also corrected it in the methods section the manuscript, i.e., in Table 2 and in the secondary clinical assessment sub-section to replace the Grasp and Release Test by the Prehension subtest of the GRASSP.

R2.2. The planned stratification by age (three categories), sex, and AIS grade may generate a large number of strata relative to the proposed sample size (n=40). This could result in sparse strata and reduce the effectiveness of the randomization procedure. The authors should provide a rationale for this approach or consider reducing the number of stratification variables**.**

Response R2.2. We agree that stratifying by age (three categories), sex, and AIS grade yields a relatively large number of potential strata compared to the total sample size (n = 40), which may lead to sparse or empty cells. However, these variables were selected a priori because they are clinically important prognostic factors known to influence outcomes in this population and could confound treatment effects if imbalanced between groups. In the text, we provided the following justification:

'Stratified randomization with a 1:1 allocation ratio will be used to ensure balance across key characteristics, including age, sex, and SCI severity as classified by the American Spinal Injury Association (ASIA). These variables were selected a priori because they are clinically important prognostic factors known to influence recovery and functional outcomes after spinal cord injury, and imbalance across groups could bias treatment effect estimates. We acknowledge that the number of potential strata is relatively large compared to the target sample size (n = 40), which may result in sparse strata. To address this, a restricted randomization approach will be used (e.g., permuted block randomization). In addition, baseline characteristics will be carefully assessed and any residual imbalance in key variables (particularly SCI severity) will be adjusted for in the primary analysis. This approach balances the need to control for important prognostic factors with the practical constraints of a small sample.'

R2.3. The proposed statistical analysis requires further clarification. Although repeated-measures ANOVA is specified as the primary analysis, the protocol also states that missing data will be handled using multiple imputation or mixed-model estimation. Given the likelihood of missing data in a longitudinal trial, a mixed-effects model may be more appropriate than repeated-measures ANOVA. The authors should clarify the primary analytical approach, the strategy for handling missing data, and whether both intention-to-treat and per-protocol analyses will be conducted.

Response R2.3: Thank you for this helpful comment. We have revised the Statistical Analysis section to clarify the primary analytical approach. Specifically, we now specify that a linear mixed-effects model will be used as the primary analysis, with fixed effects for group, time, and the group × time interaction, and a random intercept for participants to account for within-subject correlations. We also clarify that the primary analysis will follow the intention-to-treat principle, with all available observations included in the mixed-effects model, which appropriately accommodates missing data under the missing-at-random

assumption. In addition, we state that sensitivity analyses using multiple imputation may be conducted, if warranted, to assess the robustness of the findings.

VERSION 2 - REVIEW

**Reviewer**	**2**
**Name**	**Minetama, Masakazu**
**Affiliation**	**Wakayama Medical University Kihoku Hospital, Spine Care Center**
**Date**	**09-Jul-2026**
**COI**	

I do believe that the authors have addressed the issues that I raised and that the manuscript is significantly better than before. This is a nice paper and I would recommend publication.

VERSION 2 - AUTHOR RESPONSE

Reviewer: 2

Comments to the Author:

R2.1 I do believe that the authors have addressed the issues that I raised and that the manuscript is significantly better than before. This is a nice paper and I would recommend publication.

Response R2.1: We sincerely thank the reviewer for their positive evaluation of our revised manuscript and for their constructive comments throughout the review process. We appreciate the reviewer's recognition of the improvements made to the manuscript and are grateful for their recommendation for publication.
