# Supplementary material for: Combined activity-based therapy and transcutaneous spinal cord stimulation as an intensification and supplemental approach for upper limb sensorimotor recovery: a randomised controlled trial protocol for in-patients with subacute spinal cord injury
Source: BMJ Open. 2026 Aug 14;16(8):e117320. doi: 10.1136/bmjopen-2026-117320 (PMC13479523; doi:10.1136/bmjopen-2026-117320)
Supplement: Supplementary file 2 [file bmjopen-16-8-s001.docx]

# informed consent form

Participants with a spinal cord injury

1. **Study title**

Evaluate the effect of the combination of activity-based therapy and cervical spinal stimulation on the functional recovery on the upper body in people with a spinal cord injury

2. **Principal Investigators**

**Dorothy Barthélemy, pht, M.Sc., Ph.D.**

**Associate professor**, École de réadaptation, Université de Montréal

**Researcher**, Centre de recherche interdisciplinaire en réadaptation (CRIR)

Institut universitaire sur la réadaptation en déficience physique de Montréal (IURDPM) du Centre intégré universitaire de santé et de services sociaux du Centre-Sud-de-l’Ile-de-Montréal

**Diana Zidarov, PhD, MSc, pht**

**Researcher,** Centre de recherche interdisciplinaire en réadaptation (CRIR)

Institut universitaire sur la réadaptation en déficience physique de Montréal (IURDPM) du Centre intégré universitaire de santé et de services sociaux du Centre-Sud-de-l’Ile-de-Montréal

3. **Collaborators**

**Marika Demers,**

**Assistant professor**, École de réadaptation, Université de Montréal

**Researcher**, Centre de recherche interdisciplinaire en réadaptation (CRIR)

Institut universitaire sur la réadaptation en déficience physique de Montréal (IURDPM) du Centre intégré universitaire de santé et de services sociaux du Centre-Sud-de-l’Ile-de-Montréal

4. **Clinicians**

**Julie Comtois**,

Occupational therapist and project clinical lead

**Catherine Dansereau**,

Physiotherapist

**Marie-Eve Lamarche**,

Occupational therapist

**Marion Lack**,

Occupational therapist

**Sabrina Major**,

Occupational therapist

**Nancy Dubé**,

Clinical coordinator

5. **Collaborators**

Refer to appendix

6. **Funding agency**

This research project benefits from the financial support of the Lindsay funds of the Lindsay Rehabilitation Hospital (LRH) foundation. The mission of this foundation is to encourage research and excellency in the domain of rehabilitation for people with significant and long-term deficiency, to improve their autonomy and social integration.

7. **Introduction**

We are inviting you to participate in a research project. Before agreeing to participate in this project, please take the time to read and carefully consider the following information.

This consent form explains the aim of this study, the procedures, advantages, risks and inconveniences, as well as the persons to contact, if necessary.

This consent form may contain words that you do not understand. We invite you to ask any question that you consider useful to the investigator and the other staff members assigned to the research project and ask them to explain any word or information that is not clear to you.

8. **Description of the project and its objectives**

A cervical spinal cord injury (SCI) is a life-changing trauma that affects upper body function, often resulting in the loss of sensory or motor abilities. Physical rehabilitation is the conventional treatment currently used to optimize a person's functions. However, recent studies show the potential beneficial effects of combining activity-based therapy with cervical spinal stimulation on motor function.

The project aims to evaluate the impact of combining activity-based therapy with cervical spinal stimulation on upper limb motor control in individuals with SCI.

9. **Nature of participation**

Forty participants aged 16 or older with a spinal cord injury classified as AIS A to D and a neurological injury level above second thoracic vertebra will be recruited. Each participant will be randomly assigned to one of two groups: the experimental group or the control group. The experimental group will receive activity-based therapy sessions combined with cervical spinal stimulation, while the control group will receive activity-based therapy with a placebo stimulation. Your assignment to one of these two groups is randomized, which means you will not be informed of the specific intervention you will receive.

Your participation in this project includes 3 assessment sessions and 20 training sessions. The training sessions will take place over a period of 6-8 weeks on the main floor (salle Paragym) of the Institut de réadaptation Gingras-Lindsay de Montréal IRGLM (6300 Darlington Avenue).

In the first session, we will ask you to answer questions about your medical history. Your answers will allow us to ensure that you do not have any medical or physiological condition that makes you ineligible to participate in the present study. In addition, your answers will assist us in the interpretation of the results of the experimental procedures. We will ask you to wear a t-shirt to be comfortable during the training and assessment sessions.

**DETAILS OF THE STUDY:**

Figure 1

Participants will undergo 6-8 weeks of training (Figure 1), with around 3 training sessions of an hour per week, for a total of 20 trainings. Clinical and neurophysiological assessments will be done at 4 different time points: 1 week before the start of training (T1 Evaluation), one week after the last training session (T2 Evaluation), and one month after the end of treatment (T3 Evaluation).

In addition, you may be invited to take part in a one-hour interview to assess your satisfaction with the interventions during Evaluation Time 2 (T2).

a. **Training session (1h30):**

These are sessions of activity-based therapy adapted to your functional level and are done by research professionals – clinicians. Each session will include 5 parts:

- Mental imaging exercises: you will not do any movement during these exercises; you will imagine the movement while following a sequence proposed by research professionals – clinicians.
- Cardiovascular warm-up exercises: using equipment that will allow to train your cardiovascular system;
- Weight bearing and stretching exercises: exercises to prepare your upper body muscles for the strengthening exercises;
- Muscle strengthening exercises: exercises to increase your strength, with adapted equipment if necessary;
- Activity-based exercises: exercises composed of repetitive that are present in you daily life;

The session will last a total of 1h30, during which you will benefit from around 45 minutes of active therapy, including time for adjustments and transitions.

During each training session, we will place electrodes on the back of your neck to apply cervical spinal stimulation (intervention group) or a placebo stimulation (control group).

a. **Evaluation session (5h-5H30 h):**

**We are going to split the evaluation session over several days to avoid fatigue.**

1. Clinical evaluation (1h30-2h)

A clinician will conduct the clinical evaluation. You will be asked to perform a series of clinical tests to assess your muscle strength, sensations, and the quality of movements you can make with your arms. We will also ask you about your perception of effort.

.

2. Neuroplasticity evaluation
i. Sensory evaluation (1h30)

A research professional—clinician will perform the sensory evaluation. You will be seated comfortably in a chair or in your wheelchair. A nerve in your arm will be briefly stimulated by an electrode placed above the inner fold of your elbow. Small electrodes placed on your head will record the activity of neurons in your brain at the time of stimulation. This recording of brain activity is painless. Four sets of stimulations (each lasting 5 minutes), separated by 2-minute rest periods, will be conducted. These evaluations will take approximately 1 hour, including setup time.

ii. Motor evaluation (1h)

A research professional—clinician will perform the motor evaluation. You will be seated comfortably in a chair or in your wheelchair. A magnetic field will be applied by placing a figure-8 shaped device on your head (TMS), which will trigger the activation of your motor nerves, leading to muscle activation. This muscle activation will be recorded by an electrode (EMG) placed on your arm. This recording is painless. Four sets of stimulations (each lasting 5 minutes), separated by 2-minute rest periods, will be conducted. These evaluations will take approximately 1 hour, including setup time.

iii. Mapping (1h)

Transcutaneous spinal stimulation is also used to assess the transmission of electrical signals from your spinal cord to your muscles. Research professionals will apply electrical stimulation to your spinal cord and collarbone using surface electrodes to stimulate the muscles in your arms. Muscle responses will be recorded using sensors EMG placed on the skin of your arms. In addition, we will look at the parameters for stimulating the spinal cord to obtain an optimum motor response, which will enable us to determine the stimulation parameters chosen for you that we will apply during the therapies.

3. Movement kinematic evaluation (10 min)

Once a week and at each evaluation point, your movement quality during a specific task will be assessed. The task involves picking up a glass and bringing it to your mouth. This movement will be performed five times with each arm. The movement will be recorded using a video camera for further analysis.

4. Interview (1h):

You may be invited to participate in a one-hour interview to assess your satisfaction with the interventions during evaluation time 2 (T2). This interview will take place in person and will be audio recorded

10. **Personal benefits of participating in the study**

You will not personally gain any direct benefits from participating in this study. However, you may contribute to advancing knowledge in the field of treating patients with SCI. This information will be useful in guiding the treatment of these patients, which will have a positive impact on both the lives of patients and the healthcare system.

Your participation in this project gives you access to a greater number of physical rehabilitation sessions, allowing for a more intensive rehabilitation process.

11. **Risks and inconveniences associated with participating in the study**

It is understood that your participation in the study will not affect the care and services you receive or will receive from your rehabilitation institution.

**RISKS**

The known risks associated with participating in this research are as follows:

- Risk of Discomfort:

The use of electrodes and stimulation may cause some discomfort. The stimulation level will be adjusted according to your feedback and tolerance threshold. Adjustments will be made throughout the training sessions to avoid any discomfort. There is a potential risk of burns from electrical stimulation, but precautions will be taken, such as using low intensities and regularly checking the skin to prevent this from happening.

TMS (Transcranial Magnetic Stimulation) is commonly used in research and does not cause pain. Although we use low intensities, you may experience a slight headache.

Training may cause muscle soreness after the first sessions. This should gradually subside as your body adapts to the physical exertion.

- Risk of Fatigue:

The effort required during laboratory evaluation and training sessions may cause temporary fatigue. If you feel tired during any session, you can rest at any time before continuing.

- Emotional Distress:

Your participation in this project may trigger unpleasant emotions. If this happens, you can discuss it with a member of the research team, who will help you find resources for support, which may include a conversation with your treatment team.

**INCONVENIENCES**

- Transportation/Duration of Participation:

Traveling from your home to the research sites, the duration of evaluation and training sessions, and attending multiple lab sessions may be inconvenient for some participants.

If you use adapted transport, this could pose an additional challenge. Our research team will assist you as needed from the vehicle to the lab and back. If you need to wait for adapted transportation after training, there are designated waiting areas on the ground floor of the IRGLM that you can use.

- Use of Electrodes:

The placement of electrodes may require shaving hair on the areas where the electrodes will be applied. Strict hygiene protocols will be followed (single-use razors, hypoallergenic tape, cleaning the skin with alcohol). The skin where the electrodes are placed may become irritated. In such cases, a soothing lotion will be applied to your skin. If the irritation persists for more than 24 hours, please notify one of the project managers, who will assist you in addressing this issue.

- Gel Application in Hair:

During EEG evaluations to record brain activity, gel must be applied to each hole in the cap on your scalp. A research clinician will rinse your hair with water, so your hair will be wet at the end of the evaluation.

Your participation in this research project involves minimal risks for you.

12. Access to the results at the end of the research

At the end of the study, do you want to have access to the general results of this research project.

Yes ☐	email or address : _______________________________________

No ☐

13. **access to your medical record**

You authorize the research team to review your rehabilitation records in order to collect information related to your SCI that is necessary for the research project, including your age, the level of your injury, your medical history (such as epilepsy or a pacemaker, which could be contraindications for cortical stimulation), any fractures, and your ASIA classification.

14. **Confidentiality**

All personal information collected concerning you during the study will be coded to ensure its confidentiality. Only the members of the research team will have access to it. However, for research project control purposes, your research record could be consulted by a person mandated by the *Comité d’éthique de la recherche en réadaptation et en déficience physique* or by the *Direction de l’éthique et de la qualité du ministère de la Santé et des Services sociaux du Québec*. This person adheres to a policy of strict confidentiality.

The research data (paper and recordings) will be kept under lock and key at the IRGLM by the person in charge of the study. Digital data will be stored on a secure server of *Centre de recherche interdisciplinaire en réadaptation du Montréal métropolitain*. All data will be kept for a period of 7 years following the end of the project, after which it will be destroyed.

In the event that the results of this study are presented or published, no information that can identify you will be included.

15. **Video recording and/or taking photographs**

It is possible that certain sessions will be recorded via audio or video or that photographs will be taken of you. We would like to use these recordings or photographs, with your permission, for the purpose of training and/or scientific presentation purposes. However, it is unnecessary to consent to this in order to participate in this project. If you refuse, the recordings and photographs concerning you will be destroyed at the end of the project to respect your confidentiality.

Do you authorize us to use your photographs or recordings for the purpose of training or scientific presentations and to keep these recordings with your research data?

Yes ☐	No ☐

16. **Voluntary participation and right of withdrawal**

You are free to accept or refuse to participate in this research project. You may withdraw from this study at any time, without having to give a reason and without suffering prejudice of any kind. You simply have to notify the resource person of the research team. In case of withdrawal on your part, the audiovisual and written documents concerning you will be destroyed at your request.

17. **Secondary use of information for research purposes**

The information you provide may be used, before the expected date of its destruction, in other research projects that will focus on the different facets of the topic for which you are solicited today. These possible projects will be the responsibility of the principal investigator and will be authorized by the *Comité d’éthique de la recherche en réadaptation et en déficience physique*. The research team is committed to maintaining and protecting the confidentiality of your data under the same conditions as for this project. Do you agree that your data can be used in this context?

☐ Yes	☐ No

18. **Subsequent studies**

It is possible that the results of this study will give rise to another research project. In this context, do you authorize the persons in charge of this project to contact you again and ask if you would like to participate in this new project? Your contact information will be kept for 10 years.

☐ No

☐ Yes

19. **Responsibility of the research team**

By accepting to participate in this study, you do not renounce any of your rights nor do you release the investigators, or the institution involved from their civil or professional responsibilities.

20. **Compensatory indemnity**

You will receive an amount of 50$ per evaluation session (a maximum of 150$) at the end of each evaluation session. This compensation is to cover time lost, travel expenses, parking, and other incurred inconveniences.

21. **Resource persons**

If you have any questions regarding the research project, if you wish to withdraw from this study or if you wish to inform the research team regarding an incident, you may contact: Dorothy Barthélemy, professor at the École de Réadaptation, at 514-340-2085 (ext 3049).

If you have any questions about your rights and recourse, or about your participation in this research project, you can contact the research ethics committee coordinator at 514-809-0230.

For these questions, you may also contact the Local Complaints Commissioner of the CIUSSS du Centre-Sud-de-l’Île-de-Montréal: Phone: 514-593-3600

22. **Consent** (If participant is a minor, to be completed by the person with parental authority)

I declare that I have read and understood this project, the nature and the scope of my participation, as well as the risks and inconveniences to which I may be exposed, as presented in this document. I have had the opportunity to ask all my questions regarding the different aspects of the study and to receive answers to these questions. A signed copy of this information and consent form must be provided to me.

I, undersigned, voluntarily accept to participate in this study. I can withdraw my participation in this study at any time without prejudice of any kind. I certify that I was allowed all the time necessary to make my decision.

Participant’s Name:	SIGNATURE

Signed at _________ on ___________, 20_____

**THE RESEARCHER MUST GIVE A SIGNED COPY OF THE CONSENT FORM TO THE PARTICIPANT AND KEEP ANOTHER ONE IN THE RECORD**

23. **Assent (to be completed if participant is a minor, section to be filed in by participant)**

I understand that I may terminate my participation in this study at any time and I understand what my participation in this study involves. I accept to participate in this study.

PARTICIPANT’S NAME	SIGNATURE

Signed at ________________, on ___________, 20_____

24. **Commitment of the investigator or her/his representative**

For adult participant:

I, undersigned, ___________________________________, certify:

(a) that I have explained to the signatory the terms of the present form;
(b) that I have answered any questions that she/he asked me in this regard;
(c) that I have clearly indicated that she/he remains, at any time, free to terminate her/his participation in the research project described above;
(d) that I will provide her/him a signed and dated copy of this form.

*________________________________*

Signature of the Lead Investigator or his representative

Signed on _________ of ___________, 20_____

For a minor participant:

I, the undersigned ___________________________ confirm that I have explained to the legal representative and to the minor mentioned above:

(a) the purpose of the research project, its advantages, disadvantages and risks

(b) the nature of participation

(c) that they have the option of withdrawing from this research project at any time

(d) that I will provide them with a signed and dated copy of this form.

Name of authorized researcher or research team member

Signature of authorized researcher or research team member

Done at _________________, on______________________ 20______.

**APPENDIX**

The collaborators implicated in this project are :

# Sukhvinder Kalsi-Ryan, pht, BScPT, M.Sc., Ph.D.

**Associate professor**, Département de physiothérapie, Université de Toronto

**Researcher** affiliated in the domain of evaluation and recovery of the upper body and the vertebral colon pathology at KITE-UHN, Lyndhurst Center

**Victoria Duda**

**Associate professor**, École d’orthophonie et d’audiologie, Université de Montréal
**Emerging researcher,** Centre de recherche interdisciplinaire en réadaptation (CRIR)

Institut universitaire sur la réadaptation en déficience physique de Montréal (IURDPM) du Centre intégré universitaire de santé et de services sociaux du Centre-Sud-de-l’Ile-de-Montréal

**Véronique Girodias,**

**Psychologist**, Institut de réadaptation de Gingras Lindsay de Montréal
